# What Is Significant in Modern Augmented Reality: A Systematic Analysis of Existing Reviews

**DOI:** 10.3390/jimaging8050145

**Published:** 2022-05-21

**Authors:** Athanasios Nikolaidis

**Affiliations:** Department of Informatics, Computer and Telecommunications Engineering, International Hellenic University, 62124 Serres, Greece; nikolaid@ihu.gr

**Keywords:** augmented reality, umbrella review, AR applications

## Abstract

Augmented reality (AR) is a field of technology that has evolved drastically during the last decades, due to its vast range of applications in everyday life. The aim of this paper is to provide researchers with an overview of what has been surveyed since 2010 in terms of AR application areas as well as in terms of its technical aspects, and to discuss the extent to which both application areas and technical aspects have been covered, as well as to examine whether one can extract useful evidence of what aspects have not been covered adequately and whether it is possible to define common taxonomy criteria for performing AR reviews in the future. To this end, a search with inclusion and exclusion criteria has been performed in the Scopus database, producing a representative set of 47 reviews, covering the years from 2010 onwards. A proper taxonomy of the results is introduced, and the findings reveal, among others, the lack of AR application reviews covering all suggested criteria.

## 1. Introduction

Augmented Reality (AR) is a technological field that has already fueled diverse application areas for a few decades. A typical definition of AR can be found in [1]: “Augmented reality is a system that enhances the real world by superimposing computer-generated information on top of it”. If we would like to search for the first appearance of the term, we should go back to 1992 and the paper by Caudell et al. [2]. In this specific publication, the authors present a head mounted display (HMD) that is used to aid the aircraft manufacturing process.

Ever since that publication, there has been a thriving increase of works in several application areas where AR could be utilized. The first known paper that surveys the state-of-the-art in AR applications is [3] by Azuma. The author already recognized, at this early stage, six different application classes: medical, manufacturing and repair, annotation and visualization, robot path planning, entertainment, and military aircraft. The works referenced span the years range of 1989–1997, and the author describes the main characteristics of these systems, as well as the problems that the up-to-date systems faced in terms of registration and sensing.

**Figure 1:**
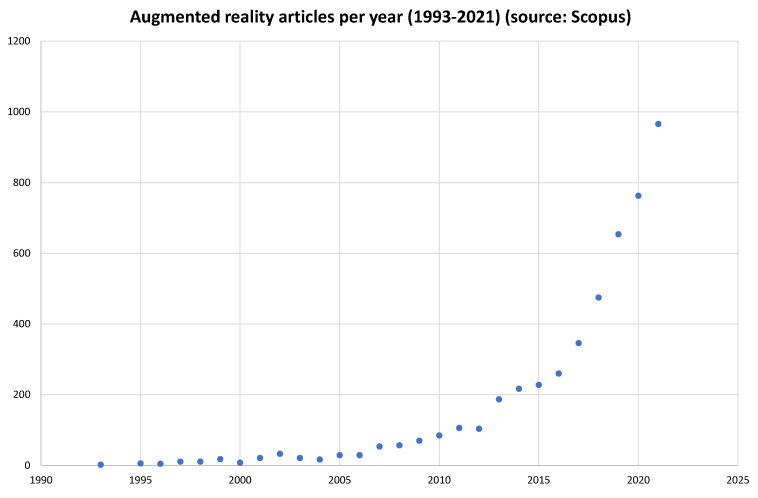
Scopus articles referring to augmented reality per year (1993–2021).

In the latest years, there has been an apparent rise in published material concerning AR as one can see in Figure 1, where the Scopus database was searched for journal papers containing the words “augmented” and “reality” in their title, without any further screening.

**Figure 2:**
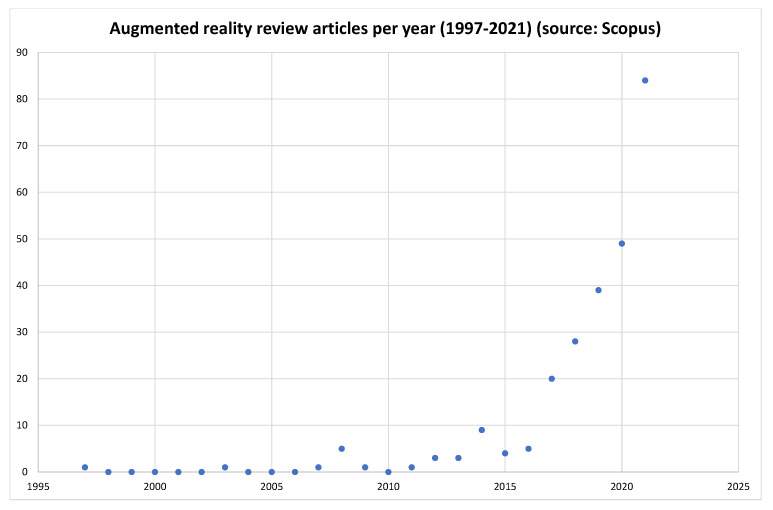
Scopus review articles referring to augmented reality per year (1997–2021).

As the years advanced, there were even more application fields that found support in AR, such as education, tourism, retail, and geoscience. In each of these fields, many new findings were published. Researchers had to find their way into exploring the current state-of-the-art in each different area, depending on the specific AR application they were aiming at. Thus, reviews of specific AR applications areas, as well as reviews of specific technical aspects of AR, started to emerge. In Figure 2 we can see the annual trend of journal articles found in Scopus database, containing the words “augmented”, “reality”, and “review” or “survey” in their title, without any subsequent screening.

The increased interest both for AR itself, as well as for surveying its application areas and technical aspects, rendered it crucial, in our mind, to perform a review of what areas have attracted more interest by the researchers, measured by the number of reviews in each area and the extent to which the application characteristics have been analyzed. As a result, the areas which deserve to be further considered by researchers for AR application development will be identified, as well as the weaknesses that existing AR systems reveal in practice, in order to suggest future research directions. In order to do so, a sort of “umbrella” review will be performed, in the sense that existing reviews in all AR application areas will be covered. The only similar attempt made before was that by Nesenbergs et al. [4], but it only focused on the specific application area of remote higher education also covering Virtual Reality (VR). Another point that should be stressed is that no attention was paid to the specific query that has been executed at each instance and each database, presuming that the authors performing the survey have a broader knowledge of the scientific area and, thus, perform an acceptable search.

The research questions that are going to be answered based on the results are the following:

- **RQ1:** Are all application areas adequately covered by current AR reviews?
- **RQ2:** Are all technical aspects of proposed AR systems covered by AR reviews?
- **RQ3:** Is it possible to establish common taxonomy criteria for surveying any AR applications area?
- **RQ4:** Is it possible to recognize which technical aspects of AR are considered more significant depending on the AR application area?

The layout of the rest of the paper is as follows. In Section 2 a brief retrospect of publications conducting AR reviews, but not focusing on a particular application area, is presented. In Section 3, the adopted search and screening process is described in detail, in order to end up with the set of review papers that are going to be analyzed. In Section 4, the selected taxonomy criteria are presented, together with a brief description of them. In Section 5, our findings are presented, and a discussion on the posed research questions follows. Lastly, in Section 6, conclusions are drawn about the performed study and future directions are proposed.

## 2. Related Work

After the original review paper by Azuma [3], an incremental review was presented by Azuma et al. [5] four years later, where all the up-to-date developments in the field were considered. These included trends of that time in displays, tracking sensors, calibration, user interfaces, interaction, rendering, as well as on mobile, collaborative, and commercial applications, together with a discussion on existing problems and limitations. In 2011, Carmigniani et al. [6,7] went through all related computer vision methods, devices, interfaces, and AR systems. They also made a special mention to mobile systems and subsequently presented applications in advertising, entertainment, education, and medicine. In 2012, Berryman [8] presented a brief overview of AR history, concept, and uses, specifically mentioning marketing, entertainment, sightseeing, industry, fashion, and medicine. Mekni et al. [9] presented medical, military, manufacturing, visualization, entertainment and games, robotics, education, marketing, navigation and path planning, tourism, geospatial, urban planning, and civil engineering applications of AR. In 2017, Chatzopoulos et al. [10] focused on applications of Mobile Augmented Reality (MAR) such as tourism and navigation, entertainment and advertisement, training and education, geometry modeling and scene construction, assembly and maintenance, information assistant management, and big data driven MAR, and provided an overview of related user interfaces and user experience, system components, tracking and registration, network connectivity, data management, system performance, and sustainability and challenging problems. Cipresso et al. [11] later stressed the differences between VR and AR, and, for both technologies, identified document clusters corresponding to areas of research, as well as networks of document co-citations. Chen et al. [12] presented a brief overview of AR display, registration and interaction technologies, AR SDKs, application areas, and the AR cloud. In [13], Merino et al. discussed both technology-centric and human-centric evaluations present in Mixed Reality (MR) and AR literature. More recently, Arena et al. [14] discriminated between games, medical applications, and others briefly described the hardware, software, and design limits of AR systems, and made a reference to AR in Industry 4.0.

Apart from reviews regarding AR as a whole, several papers have reviewed developments of specific technical aspects. As far as input device technology is concerned, some good examples are papers reviewing skin-integrated vibrohaptic devices [15,16], wearable sensors and integrated functional devices [17], thermo-haptic materials and devices [18,19], elastomeric haptic devices [20], and active materials [21].

In terms of display technology, a significant effort has been made to report technological advances. Yin et al. [22], Zhan et al. [23], and Xiong et al. [24] present general reviews in this field. On the other hand, in [25], Huang et al. focus on liquid-crystal-on-silicon technology, while Xiong et al. refer to holographic optical elements [26] and planar liquid crystal polarization optics [27]. El Jamiy et al. [28] surveyed existing works on depth perception for HMDs.

Zhou et al. [29] are concerned about three different technical aspects of AR systems, namely, tracking, interaction, and display, while Bostanci et al. [30] focus solely on user tracking, and Goh et al. [31] exclusively on interaction for MAR. User experience is considered by Arifin et al. [32], and, specifically for MAR, by Irshad et al. [33].

Kurkovsky et al. [34] focus on the particularities of handheld AR in terms of navigation and tracking, content management, and usability. In [35], Qiao et al. discuss the convergence of the Web and AR, as a successor to MAR. Si-Mohammed et al. [36] present the state-of-the-art in fusing Brain–Computer interfaces with AR. Norouzi et al. [37] refer to the fusion of AR with IVA (Intelligent Virtual Agents) and the IoT (Internet of Things). From a different aspect, Lampropoulos et al. [38] discuss the merging of AR with deep learning, semantic web, and knowledge graphs.

Last but not least, a special mention should be made to surveys on collaborative AR [39,40,41,42,43,44], which has enabled the enhancement of either co-located or remote, synchronous or asynchronous, shared workspace or shared experience applications, among others.

## 3. Searching and Screening Process

In order to end up with a decent collection of survey papers in the field of AR applications, the Scopus database was chosen for searching. This specific database includes a large number of exclusively peer-reviewed journals (40,079 as of 4 April 2022) covering all fields of science over a large period of time (1970–). The search was made using the expression:

(1) $$\begin{array}{c} \mathrm{TITLE}(\mathrm{augmented}\;\mathrm{AND}\;\mathrm{reality}\;\mathrm{AND}\;(\mathrm{review}\;\mathrm{OR}\;\mathrm{survey}))\;\mathrm{AND}\\ (\mathrm{LIMIT}-\mathrm{TO}\;\left(\mathrm{PUBYEAR},2022\right)\;\mathrm{OR}\;\mathrm{LIMIT}-\mathrm{TO}\;\left(\mathrm{PUBYEAR},2021\right)\;\mathrm{OR}\\ \mathrm{LIMIT}-\mathrm{TO}\;\left(\mathrm{PUBYEAR},2020\right)\;\mathrm{OR}\;\mathrm{LIMIT}-\mathrm{TO}\;\left(\mathrm{PUBYEAR},2019\right)\;\mathrm{OR}\\ \mathrm{LIMIT}-\mathrm{TO}\;\left(\mathrm{PUBYEAR},2018\right)\;\mathrm{OR}\;\mathrm{LIMIT}-\mathrm{TO}\;\left(\mathrm{PUBYEAR},2017\right)\;\mathrm{OR}\\ \mathrm{LIMIT}-\mathrm{TO}\;\left(\mathrm{PUBYEAR},2016\right)\;\mathrm{OR}\;\mathrm{LIMIT}-\mathrm{TO}\;\left(\mathrm{PUBYEAR},2015\right)\;\mathrm{OR}\\ \mathrm{LIMIT}-\mathrm{TO}\;\left(\mathrm{PUBYEAR},2014\right)\;\mathrm{OR}\;\mathrm{LIMIT}-\mathrm{TO}\;\left(\mathrm{PUBYEAR},2013\right)\;\mathrm{OR}\\ \mathrm{LIMIT}-\mathrm{TO}\;\left(\mathrm{PUBYEAR},2012\right)\;\mathrm{OR}\;\mathrm{LIMIT}-\mathrm{TO}\;\left(\mathrm{PUBYEAR},2011\right)\;\mathrm{OR}\\ \mathrm{LIMIT}-\mathrm{TO}\;\left(\mathrm{PUBYEAR},2010)\right)\;\mathrm{AND}\\ (\mathrm{LIMIT}-\mathrm{TO}\;\left(\mathrm{DOCTYPE},‘‘\mathrm{re}’’\right)\;\mathrm{OR}\;\mathrm{LIMIT}-\mathrm{TO}\;\left(\mathrm{DOCTYPE},‘‘\mathrm{ar}’’)\right)\;\mathrm{AND}\\ (\mathrm{LIMIT}-\mathrm{TO}\;\left(\mathrm{SRCTYPE},‘‘j’’)\right) \end{array}$$

This means that:

- The title has to contain the words: “augmented”, “reality”, and either “review” or “survey”. 467 papers were originally returned as hits.
- The search is limited to a year range of 2010–2022 so that relatively recent results are also taken into account by the review paper (20 papers removed).
- Results were limited once again by demanding the document type to be designated as “article” or “review”. Sometimes, the item is characterized as a research article by the publisher, although it clearly contains a review (153 papers removed).
- The results are, subsequently, limited to source type of “journal”: review papers are traditionally lengthy and do not normally fit to a conference (2 papers removed).

**Figure 3:**
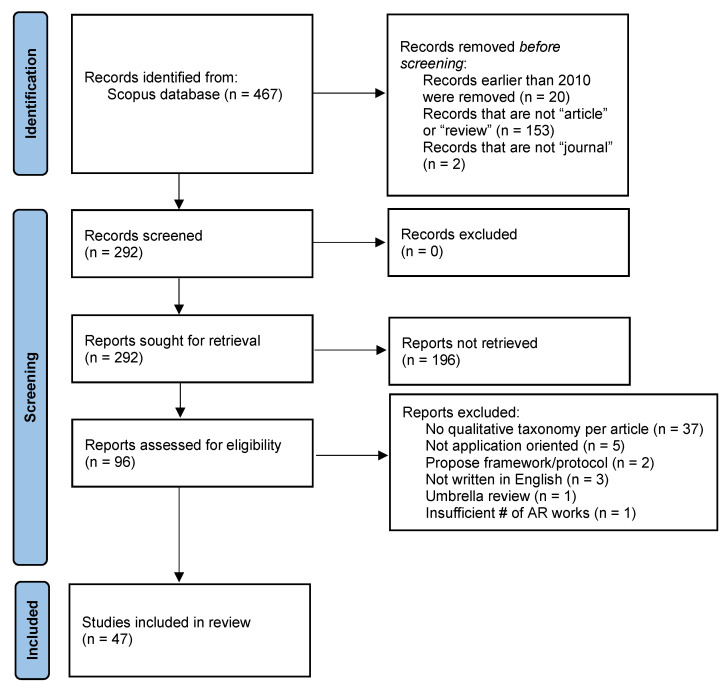
Flow diagram of the article selection process (initially: 467, finally 47).

Other commercial databases, such as Web of Science, were not considered due to access limitations through the author’s institution. Free ones such as Google Scholar were also not utilized due to their automatic mechanism of item inclusion [45]. Thus, it was found to be proper to limit the search to Scopus alone, although there may be a chance that the results are biased due to the expert inclusion criteria. The PRISMA flowchart [46] was adopted and is as shown in Figure 3.

The remaining 292 papers, resulting after the above-referenced exclusion process, were all sought for downloading via the Scopus Document Download Manager, and only 96 of them could eventually be retrieved. The subsequent manual screening process was based on the following criteria:

- **Qualitative taxonomy per article:** The review paper should contain a taxonomy of AR application works in tabular format, based on several criteria. A researcher needs to be able to pinpoint all necessary technical aspects of an existing AR application field in order to be able to easily identify strengths and weaknesses of existing methods and propose ones with real novelty. Based on this, 37 articles were excluded for not presenting such a taxonomy.
- **Application oriented reviews:** Only reviews focusing on a specific AR application field are sought, and not ones focusing on specific technical aspects of applications. 5 articles were excluded by this criterion.
- **No framework/protocol:** No existing frameworks or protocols for performing reviews are to be assessed. This way, 2 articles were excluded.
- **English language:** Only articles written in English were considered, as it is the most common and accessible language to the great majority of researchers. Three (3) articles were excluded because they were written in Spanish.
- **No umbrella reviews:** The present work can be considered an “umbrella” review, in the sense that it covers other review works. However, it probably is the first one to conduct such an analysis. In fact, only a single such article was found, and it was only covering a broader application area, not all possible AR application areas, as in the present case.
- **Sufficient number of reviewed AR works:** Since, sometimes, reviews cover also other technological areas such as VR together with AR, it is possible that some reviews do not cover a sufficient number of AR application works, and their taxonomy is thus not tailored to AR applications. This way, one review paper was excluded, which only referenced a single AR application.

After this careful screening, the remaining 47 articles underwent the subsequent analysis.

## 4. Criteria Selection and Definitions

The 47 papers that were eventually selected for reviewing contained several taxonomy criteria. After a careful study, it was concluded that there are several criteria appearing in different works that could be useful for reference by future research works. It should also be noted that the criteria names vary from one article to another. Another point is that no criteria that were not deemed as useful for drawing conclusions were taken into account, such as publication name, authors’ country of origin, or participants’ ages. In the following, each of the selected criteria is presented and defined, using a standard name each time.

### 4.1. Hardware

The specific criterion basically refers to the display type employed. Most commonly encountered types are [47]:

- **Head-worn:** The (either video or optical) see-through display is very close to the user’s eyes, since it is attached to their head (e.g., HMD). Probably the most expensive technology, but the hands are left free for interaction with the surrounding environment.
- **Hand-held:** The users hold the see-through display in their hands. This technology acts like a magnifying lens and its cost is definitely lower than that of head-worn displays, but at least one hand is occupied.
- **Spatial:** The display is positioned at a specific location and it is either video, optical, or projective. Usually, spatial displays are intended for applications with minimum interaction (e.g., HUD in military aircrafts).

### 4.2. Field of Interest

This criterion refers to the use case of the application and its description varies, depending on the specific area. For example, in [48], which is concerned about orthopedic surgery, possible fields of interest are placement, osteotomies, tumor surgery, trauma, and training/education (called “categories” in the specific paper). In the same fashion, in [49], concerning cultural heritage studies, the possible fields are museums, cultural heritage sites, and art galleries.

### 4.3. Method

This refers to the kind of approach followed by authors to conduct their research, such as pilot study, qualitative study, quantitative study, prototype description (such as in [50] concerned about healthcare), or simulation-based game, collaborative learning simulator, or inquiry-based simulator (such as in [51] in the context of education).

### 4.4. Aim

Otherwise, the objective or purpose of the work under concern is another area-specific criterion. For example, in healthcare-oriented papers the aim could be preoperative, intraoperative, or training (as in [52], concerning orthopedic surgery). In education-oriented reviews the purpose can be, for example, the preliminary evaluation of a procedure training application, or the comparison of a didactic aid based on AR with images and video [53].

### 4.5. Main Outcomes

The main results concluding the specific research. Another area-specific descriptive criterion shows, for example, that the patient’s balance and walking speed improved or that their muscle strength increased significantly [54], or that using AR can enhance mobile and remote learning, or that it can enhance students’ focus and attentiveness [55].

### 4.6. Sample

The number of participants that took part in the experiments. This may refer, for example, to patients as far as healthcare applications are concerned, or students when talking about education applications.

### 4.7. Software

This could refer either to a SDK for developing AR applications (ARToolKit, ARCore, Vuforia Engine, ARKit, Augment, etc.), a game engine (e.g., Unity), a 3D graphics API (e.g., OpenGL) or, simply, the language in which the application was developed from scratch (e.g., C++, Java, Python). The software implements all interaction, visualization, and registration tasks.

### 4.8. Tracking

This refers to the way the presentation of the AR content is triggered. A common categorization of tracking techniques is [56]:

- **Magnetic tracking:** A device that bases its function in magnetic field properties is used to calculate the position of a receiver with respect to a transmitter.
- **Vision-based tracking:** An optical sensor is employed in order to decide on the pose of the viewer. Based on the electromagnetic spectrum range and the object dimensionality utilized, a further categorization can be introduced:
  - **Infrared tracking:** Infrared light sources (LEDs) are, usually, attached to the target of interest and a proper camera in the environment receives the emitted light (configuration can be inverted with respect the LED and camera positions).
  - **Visible Light Tracking:** The most common optical sensor type, found practically in any consumer electronic device (cameras in smartphones, tablets, laptops, and so on) receives visible light from the environment. Another level of taxonomy can be introduced, based on what kind of feature is the visual trigger:
    * **Fiducial or marker-based tracking:** A static planar image (marker) attached to specific targets is required to activate the augmented content. Examples are QR codes, logos, and product packaging. In this case, the virtual content is anchored to the marker (it is displayed in a specific location with regard to the marker).
    * **Natural feature or markerless tracking:** Instead of resorting to a marker, these techniques scan the whole environment for naturally existing features that are unique and might trigger the superposition of the virtual items. It is preferable that the natural image contains enough edges and corners to make it easier to be recognized.
    * **Model based tracking:** A 3D object model instead of a planar marker is used to trigger the augmentation.
  - **3D structure tracking:** This category is based on creating range images, usually by means of a pair of an infrared projector and an infrared sensor (e.g., Kinect). Such a device can perform full 3D reconstruction of a scene.
- **Inertial tracking:** Sensors such as gyroscopes and accelerometers are employed in order to measure all three angles of rotation of the object being tracked, as well as its change of position.
- **GPS or Location-based tracking:** This trigger type is simply based on current geographic coordinates, rendering such techniques suitable for wide area applications, such as those demanding directional guidance.
- **Hybrid tracking:** As one expects, this category fuses input from different kinds of sensors in order to improve tracking performance and overcome problems of specific sensor types.

### 4.9. Limitations

This criterion concerns the weaknesses of the proposed work. For example, in an image-guided therapy system [57], a drawback would be having to attach a marker to the bone. In an educational context [58], a limitation would be the low graphics processing power in mobile devices.

### 4.10. Modalities

Modalities refer to the different input or output sensory channels for human–computer interaction [59]:

- **Input modalities:** Channels originating from humans and destined at computers:
  - **Vision:** For example, when the camera tracks an AR marker.
  - **Tactility:** Clicking a mouse button is an example.
  - **Audition:** e.g., a voice command.
  - **Kinesthetics:** For example, sensing the position or movement of hands.
- **Output modalities:** Channels originating from computers and destined at humans:
  - **Vision:** For example, presentation of 3D graphics on a screen.
  - **Audition:** e.g., sound effects accompanying a visual augmented item.

## 5. Results and Discussion

### 5.1. AR Application Areas Coverage

**Figure 4:**
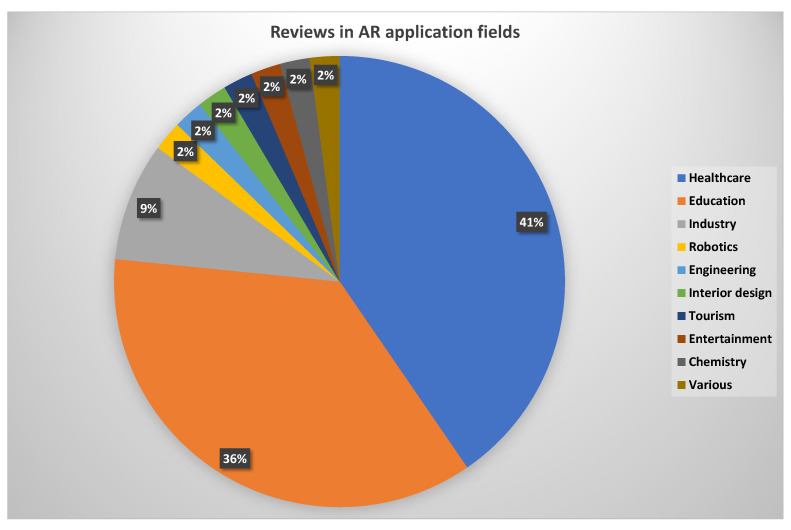
Distribution of review articles over application fields.

Since the gathered papers concerned reviews of specific AR application areas, the first thing that comes into question is how these articles are distributed with respect to application field. The pie chart in Figure 4 reveals the fact that the lion’s share belongs to the areas of healthcare and education, scoring 41% (19 papers) and 36% (17 papers), respectively. This indeed comes as no surprise, since these two are the main branches of the social sector. Next comes the field of industrial applications with 9% (4 papers), an area that has attracted a lot of attention since the very beginning of AR. The rest of the areas that have been encountered after the aforementioned searching and screening process are robotics, engineering, interior design, tourism, entertainment, and chemistry, all having the same minimum percentage of 2% (1 paper). Lastly, a single paper by Parekh et al. [60] addressing three different areas, namely healthcare, entertainment, and retail, has been categorized under the label “various”, thus producing the remaining 2% of the pie chart. Since this paper does not focus on a single field, it is going to be considered in the subsequent analysis of all three application fields. It should be noted here that the absence of other well known fields of AR applications, such as military, collaborative, or geospatial, in this distribution, does not suggest that no reviews in the specific fields exist, but that they did not qualify for being further analyzed.

### 5.2. Healthcare

Starting with healthcare, which regards 41% of total articles, or 19 out of 47 (plus the one mentioned above), it should be first stressed that, under the term “healthcare”, several subcategories are considered, such as surgery, nursing, dentistry, rehabilitation, physical therapy, emergency medicine, and so on. In Table 1, a taxonomy of the 19+1 healthcare reviews is presented, based on criteria appearing in any of them.

It is evident from Table 1 that there is not a single review article employing all ten taxonomy criteria. The one being closer to performing a complete taxonomy is the one by Viglialoro et al. [61], with 8 out of 10, omitting only software and limitations in their tabular taxonomy. This finding suggests there is room for a thorough review in healthcare applications of AR that would provide a full taxonomy in tabular format for assisting future researchers in their work.

**Figure 5:**
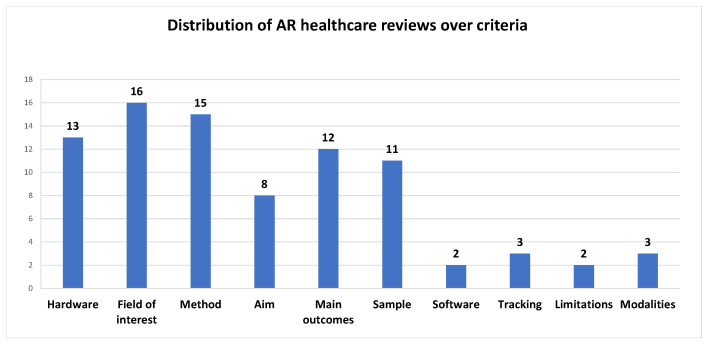
Distribution of AR healthcare reviews over criteria (min: 2–max: 16).

In Figure 5 we can see which criteria are most and which are least used by researchers in reviews of AR in healthcare.

**Table 1:** Criteria for healthcare-oriented AR review articles.

Article	Hardware	Field of Interest	Method	Aim	Main Outcomes	Sample	Software	Tracking	Limitations	Modalities
Jud et al. [48]	✓	✓	✗	✗	✗	✗	✗	✗	✗	✗
Wüller et al. [50]	✓	✓	✓	✓	✓	✗	✗	✗	✗	✗
Longo et al. [52]	✗	✓	✓	✓	✓	✓	✗	✗	✗	✗
Farronato et al. [62]	✓	✓	✓	✗	✓	✓	✓	✗	✗	✗
Arjomandi Rad et al. [63]	✗	✗	✓	✗	✓	✓	✗	✗	✗	✗
Dechsling et al. [64]	✓	✗	✓	✓	✗	✓	✗	✗	✗	✗
Guha et al. [65]	✓	✓	✗	✗	✓	✓	✗	✗	✗	✗
Casari et al. [66]	✓	✓	✗	✗	✓	✗	✗	✓	✗	✗
Gil et al. [54]	✗	✓	✓	✗	✓	✓	✗	✗	✗	✗
Burström et al. [67]	✓	✓	✓	✗	✗	✗	✗	✓	✗	✗
Almurashi et al. [68]	✓	✓	✓	✓	✗	✗	✗	✗	✓	✗
Zhao et al. [57]	✗	✓	✓	✗	✗	✗	✗	✗	✓	✗
Berenguer et al. [69]	✗	✗	✓	✓	✓	✓	✗	✗	✗	✓
Lian et al. [70]	✓	✗	✓	✓	✓	✓	✗	✗	✗	✗
Viglialoro et al. [61]	✓	✓	✓	✓	✓	✓	✗	✓	✗	✓
Parekh et al. [60]	✓	✓	✗	✗	✓	✗	✗	✗	✗	✗
Cavus et al. [71]	✓	✓	✓	✗	✗	✓	✓	✗	✗	✗
Cavalcanti et al. [72]	✗	✓	✓	✗	✗	✓	✗	✗	✗	✗
Kim et al. [73]	✓	✓	✗	✗	✗	✗	✗	✗	✗	✗
Butz et al. [74]	✗	✓	✓	✓	✓	✗	✗	✗	✗	✓

We can see that researchers in this area tend to pay more attention to the field of interest (targeted application), the method employed to perform the research, and the hardware (mostly display type) in use. On the contrary, software, limitations, tracking, and modalities seem to attract reviewers’ attention less.

### 5.3. Education

Second in order of interest to researchers comes the field of education in all its forms, including professional training and learning. Seventeen out of forty-seven surveys, a significant 36%, refer to this application area. In Table 2, a taxonomy of the 17 education-oriented reviews is presented, based on criteria appearing in any of them.

Again, there is no article to score 10 out of 10 in presenting a clear tabular taxonomy based on all selected criteria. The one closest to achieving it is the review paper by Ajit et al. [51], with a score of 9 out of 10, leaving the sample criterion behind. According to the presented taxonomy requirements, a complete review paper should address these criterion as well, since it is significant for measuring the objectivity of the study.

**Figure 6:**
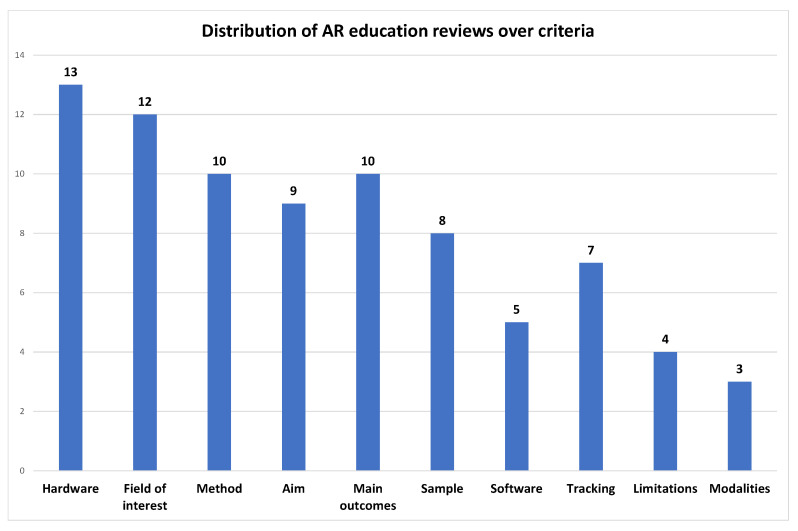
Distribution of AR education reviews over criteria (min: 3–max: 13).

In Figure 6 we can see which criteria are most and which are least used by researchers in reviews of AR in education.

Two of the three top criteria found in healthcare oriented reviews are also among the first three here: hardware and field of interest. However, in the third position we find the method and the main outcomes, which are descriptive fields capturing a great percent of the essence of any work. Modalities, limitations, software, and tracking are still at the lower end of reviewers’ preferences.

### 5.4. Other Areas

Although, in reality, all other application areas could prove equally important for assessing AR usage in them, as it was done with healthcare and education, the findings after the inclusion and exclusion process described above were too few to perform a quantitative analysis per criterion. For this reason, it was decided to consider all of them together in the current subsection. For convenience, a column was added, showing the specific application area to which each paper belongs, as one can see in Table 3.

**Figure 7:**
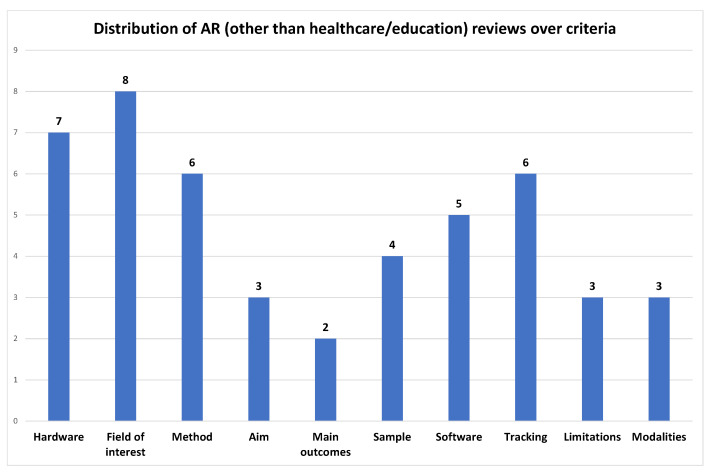
Distribution of AR education reviews over criteria (min: 2–max: 8).

Although, in this case, there is increased diversity in the nature of applications, since they come from different areas, the ones achieving the highest score in providing the selected taxonomy criteria are the publications by Li et al. [87] in the field of engineering, and the one by Ho et al. [88] in the field of industry, both with a 7 out of 10. In Figure 7 we can see which criteria are most and which are least used by researchers in reviews of AR in education.

Again, it is obvious that the field of interest and the hardware are the two of the three top trends in criteria selection by previous authors, together with tracking, which shares third place with method. To no surprise, the tracking method is referenced by reviews in the fields of industry, engineering, and robotics, which have a tendency to provide more technical details. In the last four places, we find the main outcomes, aim, limitations, and modalities.

**Table 3:** Criteria for AR review articles oriented towards other areas.

Article	Area	Hardware	Field of Interest	Method	Aim	Main Outcomes	Sample	Software	Tracking	Limitations	Modalities
Koh et al. [89]	Industry	✗	✗	✗	✗	✗	✗	✗	✓	✓	✗
Liang et al. [90]	Tourism	✗	✓	✓	✗	✗	✓	✗	✗	✗	✗
Nizam et al. [91]	Interior design	✓	✓	✗	✗	✗	✗	✗	✗	✗	✓
Li et al. [87]	Engineering	✓	✓	✓	✓	✗	✗	✓	✓	✓	✗
Makhataeva et al. [92]	Robotics	✓	✓	✗	✗	✗	✗	✓	✓	✓	✗
Ho et al. [88]	Industry	✓	✓	✓	✗	✓	✓	✓	✓	✗	✗
Boboc et al. [75]	Industry	✓	✗	✓	✗	✗	✓	✓	✓	✗	✓
Parekh et al. [60]	Entertainment / Retail	✗	✓	✗	✗	✓	✗	✗	✗	✗	✗
Fombona-Pascual et al. [93]	Chemistry	✗	✗	✗	✗	✗	✗	✓	✗	✗	✗
Costa et al. [94]	Industry	✓	✓	✓	✓	✗	✗	✗	✓	✗	✓
Marto et al. [95]	Entertainment	✓	✓	✓	✓	✗	✓	✗	✗	✗	✗

### 5.5. Answers to Research Questions

After all the above-described analysis, we are ready to answer the research questions posed in Section 1:

*RQ1: Are all application areas adequately covered by current AR reviews?*: The answer is obviously negative. Even in the most widely covered application areas, such as healthcare and education, there is no single review paper that covers all taxonomy criteria that were considered to be significant for including. At this point, it should be noted once again that examined review papers were expected to compare existing works with criteria being presented in a tabular format, so that future researchers can follow the state-of-the-art easily. Some of the selected criteria may be descriptive by nature (e.g., method, aim, main outcomes, etc.) but an experienced researcher can capture their essence in a few words. Some of the papers not selected after screening may contain useful comparison information in a totally textual format, making it difficult for the reader to come up with a conclusion about the pros and cons of the referenced works. Eventually, there are certain areas apart from all the ones encountered in the selected papers which deserve more attention. In fact, there are works proposing reviews in the field of e-commerce [96], architecture [97], construction [98], or human resource management (HRM) [99] that do not follow a systematic taxonomy such as the one proposed in the present paper.

*RQ2: Are all technical aspects of proposed AR systems covered by AR reviews?*: Although the focus of each researcher performing a review may be on different technical details of AR technology, it is apparent, from the present study, that certain aspects are considered less significant by different authors, depending on their scientific background. For example, the study by Liang et al. [90] concerning tourism does not at all refer to hardware, software, tracking, and modalities, which comprise the technical body of each application, but the authors come from a School of Hospitality, Food, and Tourism Management, and there is no coauthor from, e.g., a Computer Science Department. As another example, Longo et al. [52] present a review paper in healthcare applications of AR without any reference to all the above four criteria, but they come from a Department of Orthopedic and Trauma Surgery and a Research Unit Nursing Science.

*RQ3: Is it possible to establish common taxonomy criteria for surveying any AR applications area?*: As one can conclude from the results presented in the previous subsections, it is possible to apply all ten selected criteria to create a taxonomy of applications in any scientific area employing AR technology, since hardware, software, tracking, and modalities are technical details specific to AR, and all the rest (field of interest, method, aim, main outcomes, sample, and limitations) are general criteria that could be practically used in any case study, irrespective of technology (all six are descriptive, except for sample). These ten criteria comprise a set that enables reviewers to perform a taxonomy that will assist fellow scientists in proposing novelties in the field of AR.

*RQ4: Is it possible to recognize which technical aspects of AR are considered more significant depending on the AR application area?*: In the present paper, it is claimed that there is a degree of correlation between the popularity of a technical aspect of AR as a criterion in existing AR application area reviews and the extent to which this technical aspect is important to authors for the particular application. Indeed, it is evident from the results presented in the previous subsections that the display component is the most popular as a taxonomy criterion for authors conducting a survey in healthcare and education applications, in which the optical stimulus is the most important for the outcome of the AR use, whether the end-user is a patient, a healthcare specialist, or a student. Other aspects that drew authors’ attention to a lesser extent are software, tracking, and modalities. One reason for this may be that, in certain areas, such as healthcare and education, standard (not custom) software (e.g., ARToolKit or Vuforia/Unity), or a standard tracking method (e.g., marker-based tracking), or standard modalities (e.g., vision both for input and output) is usually employed and, thus, the authors may sometimes consider the specific criteria as redundant for taxonomy. Another reason could be that the reviewers come from a scientific area different than Computer Science and, thus, are not interested in exploring the technical aspects of the method, rather than its social implications.

## 6. Conclusions and Future Work

In the present umbrella review, it was first attempted to select a representative set of published AR application reviews, after careful screening based on specific eligibility conditions. Afterwards, a set of ten taxonomy criteria was presented that can be used in future reviews in the area. The findings were subsequently presented with respect to the selected criteria, and a further analysis of them followed in the form of answers to four specific research questions. It was thus deduced that no AR application area is fully covered until today according to the proposed taxonomy, and that the technical aspects of employed AR technology are sometimes neglected by the authors. Additionally, it was proved that the ten taxonomy criteria that were selected can be utilized in any AR application area and provide a complete picture of the state-of-the-art. Lastly, it was found out that the hardware components are deemed a more significant technical aspect by reviewers compared to software, tracking, and modalities.

It is the strong belief of the author that the present work will help future review authors in the area to conduct their surveys in a way that will assist researchers to identify the strengths and weaknesses of previous methods and introduce technical novelties.

Regarding future work, it would be a good idea to perform umbrella reviews per AR application area in order to identify potential lack of proper technical coverage in area reviews. Another point would be to propose a more fine-grained taxonomy that would reveal which previously employed AR systems perform better in practice, and thus decide which research directions would be more fruitful.

## Figures and Tables

**Table 2:** Criteria for education-oriented AR review articles.

Article	Hardware	Field of Interest	Method	Aim	Main Outcomes	Sample	Software	Tracking	Limitations	Modalities
Saidin et al. [75]	✓	✓	✗	✓	✗	✗	✗	✗	✗	✗
Gerup et al. [53]	✓	✗	✓	✓	✓	✓	✗	✓	✗	✗
Papakostas et al. [76]	✓	✗	✗	✗	✗	✗	✓	✓	✗	✓
Laine [77]	✗	✗	✗	✗	✗	✗	✓	✓	✗	✓
Vargas et al. [49]	✓	✓	✗	✗	✗	✗	✗	✓	✗	✗
Velazquez et al. [58]	✓	✓	✓	✓	✓	✓	✓	✗	✓	✗
Bui et al. [78]	✓	✗	✓	✓	✓	✓	✗	✗	✗	✗
Bölek et al. [79]	✓	✓	✗	✗	✗	✓	✗	✗	✗	✗
Challenor et al. [80]	✓	✓	✗	✓	✗	✗	✗	✓	✗	✗
Majeed et al. [81]	✗	✓	✓	✗	✓	✗	✗	✗	✗	✗
Iqbal et al. [82]	✓	✓	✗	✗	✓	✗	✗	✗	✓	✗
Rodríguez-Abad et al. [83]	✓	✓	✓	✓	✓	✓	✓	✓	✗	✗
Ajit et al. [51]	✓	✓	✓	✓	✓	✗	✓	✓	✓	✓
Alzahrani [55]	✗	✗	✓	✓	✓	✓	✗	✗	✓	✗
Barteit et al. [84]	✓	✓	✓	✓	✓	✓	✗	✗	✗	✗
Özçelik et al. [85]	✗	✓	✓	✗	✓	✓	✗	✗	✗	✗
Fernández-Batanero et al. [86]	✓	✓	✓	✗	✗	✗	✗	✗	✗	✗

## Data Availability

Data is contained within the article.

## Abbreviations

The following abbreviations are used in this manuscript:

AR	Augmented Reality
MAR	Mobile Augmented Reality
VR	Virtual Reality
MR	Mixed Reality
HMD	Head-Mounted Display
HUD	Heads-Up Display
SDK	Software Development Kit
API	Application Programming Interface
HRM	Human Resource Management
